# Gene Expression Dynamics Underlying Muscle Aging in the Hawk Moth *Manduca sexta*

**DOI:** 10.3390/genes16111306

**Published:** 2025-11-01

**Authors:** Avery Del Grosso, Beate Wone, Connor McMahon, Hallie Downs, Bernard W. M. Wone

**Affiliations:** 1 Department of Biology, University of South Dakota, Vermillion, SD 57069, USA; 2 Department of Biomedical Engineering, University of South Dakota, Sioux Falls, SD 57107, USA; 3 Department of Psychiatry and Psychology, Mayo Clinic, Rochester, MN 55905, USA

**Keywords:** muscle aging, metabolism, aging physiology, sarcopenia, advancing age

## Abstract

Background/Objectives: Muscle aging is a complex, dynamic process that impairs overall metabolism and physiological function. The molecular mechanisms underlying declines in muscle performance and metabolic efficiency remain poorly understood, largely due to the time and resource demands of traditional model organisms. The hawk moth *Manduca sexta* offers a promising alternative, with a short adult lifespan (~10 days) and notable similarities to vertebrate muscle systems, making it well-suited for time-course molecular dissection of muscle aging. Methods: In this study, we performed high-resolution temporal analysis of muscle tissues from aging *M. sexta*, spanning the physiomuscular aging process from middle age to advanced age. Results: We observed decreased expression of genes involved in fatty acid β-oxidation, ATP synthase subunits, superoxide dismutase, glutathione S-transferases, and heat shock proteins. In contrast, genes associated with proteolysis, catabolic processes, insulin signaling, *akirin*, titin, high-affinity choline transporters, and vesicular acetylcholine transporters were increased in expression. Conclusions: These changes suggest a shift toward increased proteolysis and protein catabolism with age. Our findings support the use of *M. sexta* as a complementary model for muscle aging research. However, it remains unclear whether the observed gene expression changes are driven by intrinsic, sex-specific age-related muscle aging or confounded by potential starvation effects in older males.

## 1. Introduction

Skeletal muscle aging represents a complex biological process marked by progressive declines in muscle mass, performance, and metabolic efficiency [1]. This deterioration is driven by multiple factors, notably the accumulation of senescent cells, cells that have undergone irreversible growth arrest and are considered a hallmark of aging. These changes carry significant consequences for survival in natural environments, where optimal muscle function is critical for locomotion, foraging, and evading predators [2,3]. In humans, muscle aging correlates with increased susceptibility to a range of chronic conditions, including osteoarthritis, osteoporosis, muscular dystrophy, and cancer-associated inflammation. The prevalence of metabolic disorders such as insulin resistance, type 2 diabetes mellitus, dyslipidemia, and hypertension also rises with age [4,5,6,7,8,9]. Recent studies have begun to unravel the molecular complexity of muscle aging, revealing dynamic regulatory networks that change over time [10,11,12].

Despite these advances, many gene expression studies have focused on specific disease states, isolated molecular pathways, or binary age comparisons [13,14,15,16,17]. However, muscle aging is a progressive process, and capturing its full transcriptomic landscape requires high-resolution, time-series approaches [18,19]. While vertebrate models such as rodents and fish [20,21,22,23,24] are widely used, they are often limited by cost, lifespan, and experimental complexity. Invertebrate models like *Caenorhabditis elegans* and *Drosophila melanogaster* offer advantages in lifespan and genetic tractability [25,26], but their muscle physiology diverges significantly from that of vertebrates.

The hawk moth *Manduca sexta* presents a promising complementary model for studying muscle aging. With a short adult lifespan (~10 days), a sequenced and manipulable genome, and muscle architecture, physiology, and metabolism that closely resemble those of vertebrates, including humans, *M. sexta* enables efficient, cost-effective studies of muscle aging [27,28]. Similarly to aged mammalian skeletal muscle, the ultrastructure of aged *M. sexta* flight muscles demonstrates increased mitochondrial fusion, significant decreases in sarcomere size, and pronounced myofibrillar disorganization [28]. Notably, *M. sexta* flight muscles exhibit active thermogenesis, producing heat through metabolic activity to elevate muscle temperature prior to and during flight, a functional parallel to mammalian muscle warm-up. These muscles are also synchronous, contracting once per neural impulse, functionally analogous to vertebrate muscle contraction mechanisms [27]. Taken together, these aspects demonstrate that *M. sexta* is a suitable and reliable organism for rapid modeling of mammalian muscle aging. Moreover, RNA sequencing technologies allow for near-real-time tracking of gene expression changes across the lifespan [29,30,31]. In this study, we conducted a high-resolution temporal analysis of flight muscle transcriptomes in *M. sexta* from mid to late adulthood. This approach provides a comprehensive view of transcriptomic remodeling during muscle aging and supports the use of *M. sexta* as a complementary model for muscle aging research. By capturing the dynamic nature of gene expression across the aging process, this work contributes to a broader understanding of skeletal muscle aging and lays the foundation for future hypotheses on the molecular and regulatory mechanisms underlying dynamic age-related muscle decline.

## 2. Materials and Methods

### 2.1. Model Organism

Hawk moths (*Lepidoptera: Sphingidae: M. sexta*) were obtained from a laboratory colony maintained at the University of Arizona (Tucson, AZ, USA) and used for all experiments. Larvae were reared on an ad libitum artificial diet. Upon eclosion, adults were housed individually in 28 × 28 × 28 cm cages (BioQuip Products, Rancho Dominguez, CA, USA). All life stages were maintained under controlled environmental conditions: 16 h light/8 h dark photoperiod, 25 °C, and 60% relative humidity. Adult moths were fed ad libitum with artificial nectar (Educational Science, League City, TX, USA). To prevent mating, which can influence lifespan [32] adults were housed separately by sex.

Eclosion and death dates were recorded for each individual, and the average lifespan was calculated in full days. Based on previous studies [27], middle age was defined as Day 2 and advanced age as Day 5 post-eclosion for males, and Day 4 and Day 7 for females (Table 1). Moths were sampled across diel time and age to characterize age-related changes in flight muscle gene expression. Individuals were euthanized by decapitation, and dorso-longitudinal flight muscles were dissected beginning at middle age through advanced age during both photophase (0900 h) and scotophase (2100 h, after 1 h of activity). Eight time points were sampled per sex. Dissected flight muscles were flash-frozen in liquid nitrogen within 90 s of euthanasia, pulverized under liquid nitrogen, and stored at −80 °C until further processing [27].

### 2.2. Enzyme Spectrophotometric Assays

The enzymatic activities of β-hydroxyacyl-CoA dehydrogenase (HADH) and citrate synthase (CS) were quantified via spectrophotometric analysis by measuring changes in absorbance associated with the activity of each enzyme. Flight muscle samples from male and female *M. sexta* (*n* = 20 per sex) were assayed using Eppendorf^®^ Microplate UV-VIS 96-well plates (Eppendorf, Hauppauge, NY, USA). Each plate was organized into quadrants based on age and diel time.

For each assay, 20 mg of flight muscle tissue was homogenized in 200 μL of ice-cold homogenization buffer (100 mM phosphate, 5 mM EDTA, 0.1% Triton X-100, pH 7.2) at a 1:10 ratio. Homogenates were prepared fresh for both CS and HADH assays. Samples were evenly distributed across sex, age, and diel time, with each group consisting of five biological replicates and three technical replicates per sample. All assays were conducted within 48 h of homogenization.

CS activity was determined by measuring the rate of increase in TNB concentration at 412 nm. The reaction mixture contained 100 mM Tris-HCl, 5 mM EDTA, 0.22 mM acetyl-CoA, 0.5 mM oxaloacetate, and 0.1 mM DTNB (pH 8.0). HADH activity was assessed by monitoring the rate of NADH oxidation at 340 nm. The reaction mixture included 100 mM triethanolamine-HCl, 5 mM EDTA, 0.1 mM acetoacetyl-CoA, and 0.28 mM NADH (pH 7.0).

Two microliters of tissue homogenate were added to each well, and the final reaction volume was adjusted to 200 μL. Absorbance was measured kinetically at 39 °C for 15 min using an accuSkan GO UV/Vis Microplate Spectrophotometer (Fisherbrand, Thermo Fisher Scientific, Pittsburgh, PA, USA). Readings were taken every 10 s for 90 cycles. Data were collected using SkanIt Software for Microplate Readers (v4.1.0.43, Thermo Scientific).

Enzyme activity (U/mL) was calculated using the following equations (Table 2):

Citrate Synthase (CS):

$$\frac{U}{mL}\;CS=\frac{V\times dil}{\epsilon \;\times d\;\times a}\;\times \;A_{412}$$

β-Hydroxyacyl-CoA Dehydrogenase (HADH):

$$\frac{U}{mL}\;HADH=\frac{V\times dil}{\epsilon \;\times d\;\times a}\;\times \;A_{340}$$

One unit (U) of enzyme activity is defined as the amount of enzyme that catalyzes the conversion of 1.0 μmol of substrate per minute under the specified assay conditions.

Kinetic absorbance data were averaged across technical replicates for each biological sample. Statistical analysis was performed in RStudio v1.2.1335 (RStudio, Boston, MA, USA). Three-way ANOVA was used to assess the effects of sex, age, and diel time on CS and HADH activity. Post hoc comparisons were conducted using Tukey’s HSD test. Statistical significance was set at α ≤ 0.05.

### 2.3. RNA Extraction

A total of 48 moths (*n* = 3 per sex × age × diel time) were sampled for RNA-Seq. For each sample, 50 mg of powdered muscle tissue was transferred to a 2 mL microcentrifuge tube. One mL of TRIzol Reagent was added, and samples were homogenized by vortexing. After a 5 min incubation at room temperature, 0.2 mL of chloroform was added, vortexed, and incubated for 3 min. Samples were centrifuged at 12,000× *g* for 15 min at room temperature, and the aqueous phase (~500 μL) was transferred to a new tube.

A second chloroform extraction was performed, followed by centrifugation under the same conditions. The aqueous phase was again collected, and 500 μL of 100% 2-propanol was added. Samples were inverted to mix and incubated at room temperature for 10 min before centrifugation at 12,000× *g* for 10 min. The RNA pellet was washed twice with 500 μL of 75% ethanol, each followed by centrifugation at 8000× *g* for 10 min. Pellets were air-dried under a laminar flow hood and resuspended in 20 μL of molecular-grade water. RNA samples were stored at −80 °C until use.

### 2.4. Illumina Sequencing and Differential Gene Expression Analysis

RNA-Seq was performed on *M. sexta* flight muscle samples across sex, age, and diel time. Library preparation and sequencing were conducted by Novogene (Sacramento, CA, USA). Libraries were sequenced as 150-nt paired-end reads on the Illumina NovaSeq 6000 platform, with 18 samples per high-output flow cell across 16 flow cells, generating 20–25 million reads per sample (Table 3).

Quality control included trimming of adapters, primers, and low-quality reads. High-quality reads were mapped to the *Bombyx mori* reference genome, which is more comprehensively annotated than the *M. sexta* genome [33], due to the economic importance of *B. mori*. Read counts were generated using FeatureCounts v1.5.0-p3.

Gene expression data were cross-referenced with the NCBI database to identify annotated gene IDs. Differential gene expression (DGE) analysis was performed using the DESeq2 R package version 1.30.1 [34], which models count data using a negative binomial distribution. *p*-values were adjusted for multiple testing using the Benjamini–Hochberg method [35]. Genes with adjusted *p*-values < 0.05 were considered significantly differentially expressed. Log2 Fold Change values were reported, where a value of 1.0 indicates a twofold increase and −1.0 indicates a twofold decrease in expression.

### 2.5. Orthogonal Confirmation of RNA-Seq Data by qRT-PCR

To confirm RNA-Seq results, quantitative real-time PCR (qRT-PCR) was performed on four differentially expressed genes (DEGs) from middle aged (Day 2) and advanced aged (Day 5) male *M. sexta*. Genes were selected based on their specificity to muscle tissue and function to minimize confounding variables. The selected DEGs included *akirin*, gelsolin, kelch-like protein 5 (kelch), and titin.

Akirin is a nuclear factor involved in myogenesis and myogenic differentiation [36,37]. Gelsolin is an actin-binding protein that regulates actin filament dynamics, facilitates actin recycling following cell death, and contains a caspase-3 cleavage site associated with apoptosis and membrane blebbing [38,39]. Kelch-like protein 5 belongs to a gene family encoding proteins with kelch motifs that interact with ubiquitination complexes involved in protein degradation [40]. Titin is a giant sarcomeric protein that spans from the Z-disk to the M-line, functioning as a molecular spring to generate passive tension during sarcomere extension [41,42,43], and is also implicated in protein quality control [44]. Actin was used as the housekeeping gene for normalization.

Gene-specific primers were designed (Table 4) and synthesized by Integrated DNA Technologies (Coralville, IA, USA). cDNA synthesis and qRT-PCR were performed using the Luna^®^ Universal One-Step qRT-PCR Kit (New England Biolabs, Ipswich, MA, USA) following the manufacturer’s protocol. Each 20 µL reaction contained 2.5 µL of RNA (≥100 ng/µL), 10 µL of Luna Universal One-Step Reaction Mix, 1 µL of Luna WarmStart^®^ RT Enzyme Mix, 0.8 µL each of 100 µM forward and reverse primers, and 4.9 µL of molecular-grade water.

Each gene was tested in triplicate, with four negative controls included, for a total of *n* = 40 reactions. qRT-PCR was conducted using the QuantStudio 3 Real-Time PCR System (Applied Biosystems, Foster City, CA, USA).

### 2.6. Gene Ontology (GO) and KEGG Pathway Annotation

Functional profiling of RNA-Seq data was performed using Gene Ontology (GO) enrichment analysis via the Gene Ontology Consortium (http://geneontology.org, accessed on 1 December 2019) and pathway analysis through the Kyoto Encyclopedia of Genes and Genomes (KEGG; https://www.genome.jp/kegg/, accessed on 1 December 2019). Enriched GO terms and KEGG pathways were identified based on differentially expressed genes (DEGs) with a log_2_ fold change ≥ ±1.0 and an adjusted *p*-value (*p* adj) < 0.05.

## 3. Results

### 3.1. RNA-Seq

#### 3.1.1. Differentially Expressed Genes (DEGs) in *M. sexta* Flight Muscle Across Age Classes

**Figure 1:**
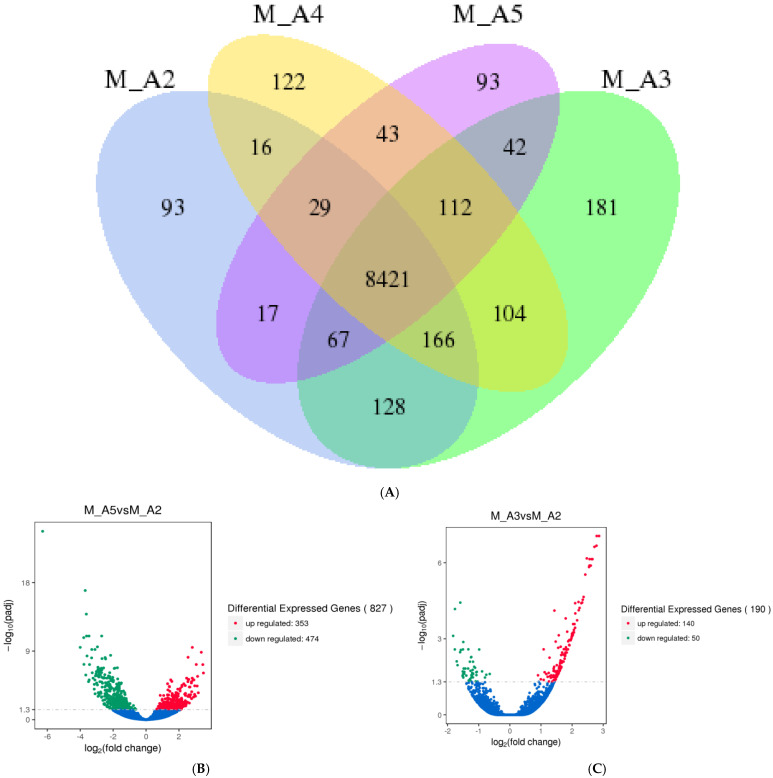

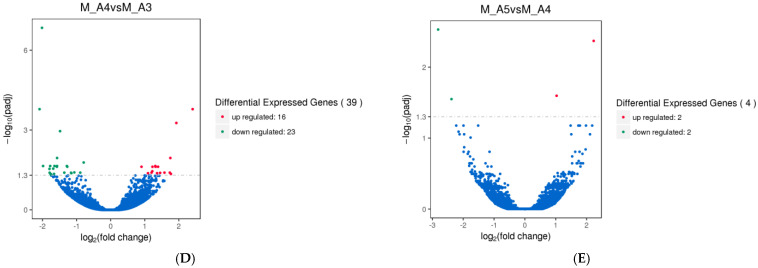
Identification and GO enrichment analysis of significantly differentially expressed mRNAs in male *Manduca sexta*. (**A**). Venn diagram showing unique and shared genes across male age classes. (**B**). Volcano plot of DEGs between Age 2 and Age 5. (**C**). Volcano plot of DEGs between Age 2 and Age 3. (**D**). Volcano plot of DEGs between Age 3 and Age 4. (**E**). Volcano plot of DEGs between Age 4 and Age 5. Red and green indicate significantly increased and decreased expression, respectively.

RNA-Seq analysis identified 8910 differentially expressed genes (DEGs) in the flight muscles of male *M. sexta* and 8946 DEGs in females. In males, 8421 genes were consistently expressed across all age classes. Additionally, 93 genes were uniquely expressed in Age 2 males, 181 in Age 3, 122 in Age 4, and 93 in Age 5 (Figure 1A). Notably, the largest shift in DEG count occurred at Age 3, indicating that the transition from Age 2 to Age 3 represents a critical period of transcriptional change.

Further analysis revealed 827 DEGs between Age 2 and Age 5 males, with 353 genes showing increased expression and 474 showing reduced expression (Figure 1B). Comparative analysis identified 190 DEGs specific to the transition from Age 2 to Age 3, including 140 with increased expression and 50 with reduced expression (Figure 1C). From Age 3 to Age 4, 39 DEGs were identified, with 15 showing increased expression and 23 showing reduced expression (Figure 1D). Only 4 DEGs were detected between Age 4 and Age 5, evenly split between increased and reduced expression (Figure 1E). These findings reinforce that the most pronounced transcriptional changes in male flight muscle occur during the transition from Age 2 to Age 3.

**Figure 2:**
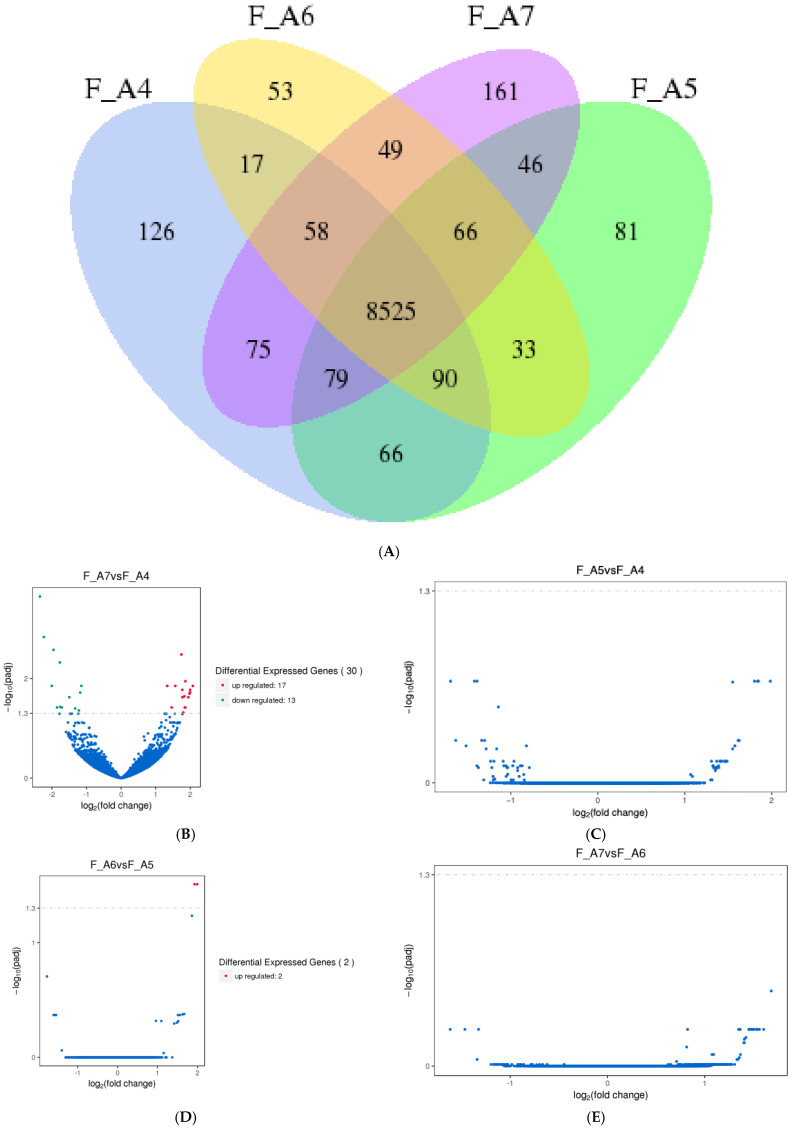
Identification and GO enrichment analysis of significantly differentially expressed mRNAs in female *Manduca sexta*. (**A**). Venn diagram showing unique and shared genes across female age classes. (**B**). Volcano plot of DEGs between Age 7 and Age 7. (**C**). Volcano plot of DEGs between Age 4 and Age 5. (**D**). Volcano plot of DEGs between Age 5 and Age 6. (**E**). Volcano plot of DEGs between Age 6 and Age 7. Red and green indicate significantly increased and decreased expression, respectively.

In females, 8525 genes were consistently expressed from middle age (Age 4) to advanced age (Age 7). Unique gene expression was observed for 126 genes at Age 4, 81 at Age 5, 53 at Age 6, and 161 at Age 7 (Figure 2A). Unlike males, female DEG patterns did not follow a clear age-related trend. Across Age 4 to Age 7, only 30 DEGs were identified (Figure 2A), with 17 showing increased expression and 13 showing reduced expression. No DEGs were detected between Age 4 and Age 5 (Figure 2C), while two DEGs with increased expression were found between Age 5 and Age 6 (Figure 2D). No DEGs were identified between Age 6 and Age 7 (Figure 2E).

**Figure 3:**
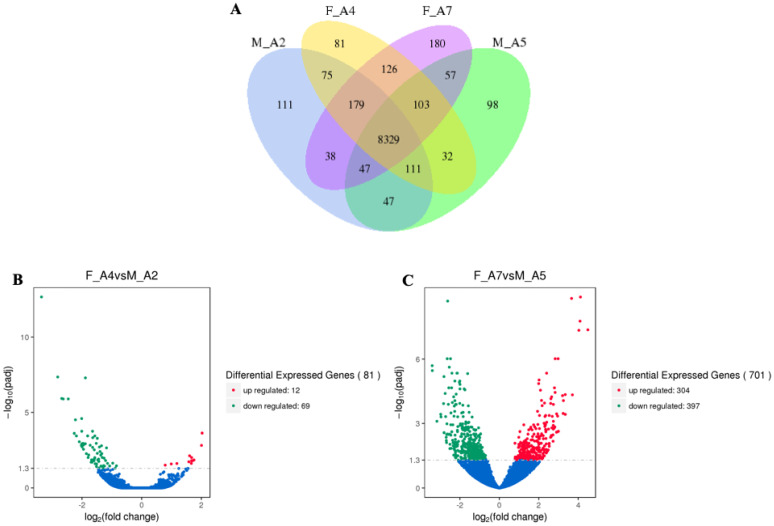
Comparative analysis of gene expression between middle age and advanced age male and female *Manduca sexta*. (**A**). Venn diagram showing overlapping and unique DEGs between sexes and age groups. (**B**). Volcano plot of DEGs between middle and advanced age. (**C**). Volcano plot of DEGs in females between middle and advanced age. Red and green indicate significantly increased and decreased expression, respectively.

Comparative analysis between middle age and advanced age males (Age 2 vs. Age 5) and females (Age 4 vs. Age 7) revealed 8329 shared genes. Unique expression was observed for 81 genes in Age 4 females, 180 in Age 7 females, 111 in Age 2 males, and 98 in Age 5 males (Figure 3A). Between Age 2 males and Age 4 females, 81 DEGs were identified, with 12 showing increased expression and 69 showing reduced expression (Figure 3B). Between Age 5 males and Age 7 females, 701 DEGs were detected, including 304 with increased expression and 397 with reduced expression (Figure 3C).

#### 3.1.2. Gene Ontology (GO) Enrichment Analysis

**Figure 4:**
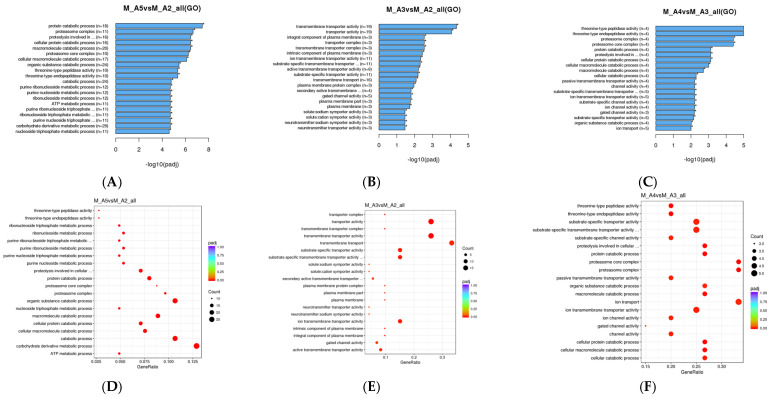
GO enrichment analysis of DEGs in male *Manduca sexta*. (**A**). Top 20 enriched GO terms comparing Age 2 and Age 5. (**B**). Top 20 enriched GO terms comparing Age 2 and Age 3. (**C**). Top 20 enriched GO terms comparing Age 3 and Age 4. (**D**). Scatter plot of enriched pathways for DEGs between Age 2 and Age 5. (**E**). Scatter plot of enriched pathways for DEGs between Age 2 and Age 3. (**F**). Scatter plot of enriched pathways for DEGs between Age 3 and Age 4. The *x*-axis represents the richness factor; the *y*-axis shows enriched pathway terms. * *p* < 0.05.

GO annotation analysis of male *M. sexta* revealed that the greatest amount of DEGs pertained to protein catabolism between middle age and advanced age moths (Figure 4A). Among male age classes, comparison between Age 2 and Age 3 moths revealed that the most DEGs were related to transmembrane transporter activity (Figure 4B). From Age 3 to Age 4, the greatest amount of significantly enriched DEGs were related to proteasomal complex proteins (Figure 4C). From Age 4 to Age 5, there was no GO enrichment data able to be produced from our results. This is likely caused by a lack of significant DEG expression changes for that age class, leading to inadequate GO identification. Interestingly, comparison of middle age to advanced age males revealed pronounced, significant changes pertaining to catabolic processes, proteasomal proteins, and proteolysis. Notably, it was not until moths progressed from Age 3 to Age 4 that there were significant changes in the transcription of catabolic processes and proteasomal elements. Comparative analysis of RNA-Seq DEGs suggested that the greatest amount of gene expression changes occurred between Age 2 and Age 3. However, GO annotation suggests that the pronounced changes between Age 2 and Age 3 are mostly changes to cellular transporters rather than direct changes to muscle structure, metabolism, and other processes commonly associated with muscle aging. The most notable changes that may directly correlate with the muscle aging phenotype appear to occur as moths transition from Age 3 to Age 4, a period marked by numerous significant shifts in protein-degradative pathways.

**Figure 5:**
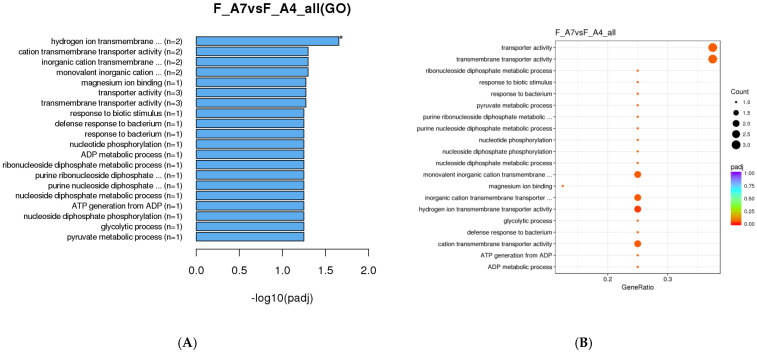
GO enrichment analysis of DEGs in female *Manduca sexta*. (**A**). Top 20 enriched GO terms comparing Age 4 and Age 7. (**B**). Scatter plot of enriched pathways for DEGs between Age 4 and Age 7. The *x*-axis represents the richness factor; the *y*-axis shows enriched pathway terms. * *p* < 0.05.

Analysis of female *M. sexta* GO annotation revealed only one significantly enriched term pertaining to hydrogen ion transmembrane transporter activity (Figure 5). Furthermore, between female age classes, no GO enrichment data were available. This result is likely due to the absence of significant DEG transcript changes among female age classes, making identification difficult.

#### 3.1.3. KEGG Enrichment Analysis

**Figure 6:**
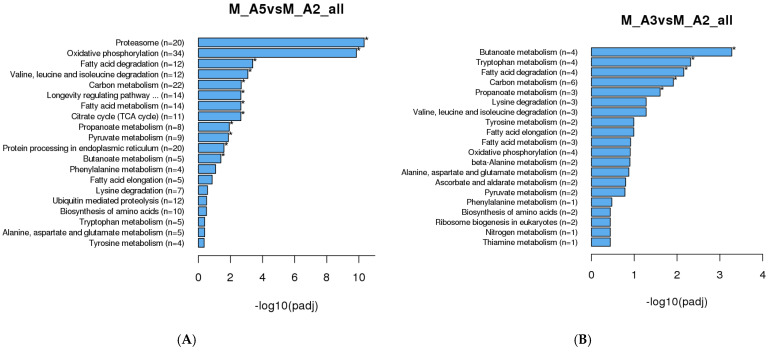

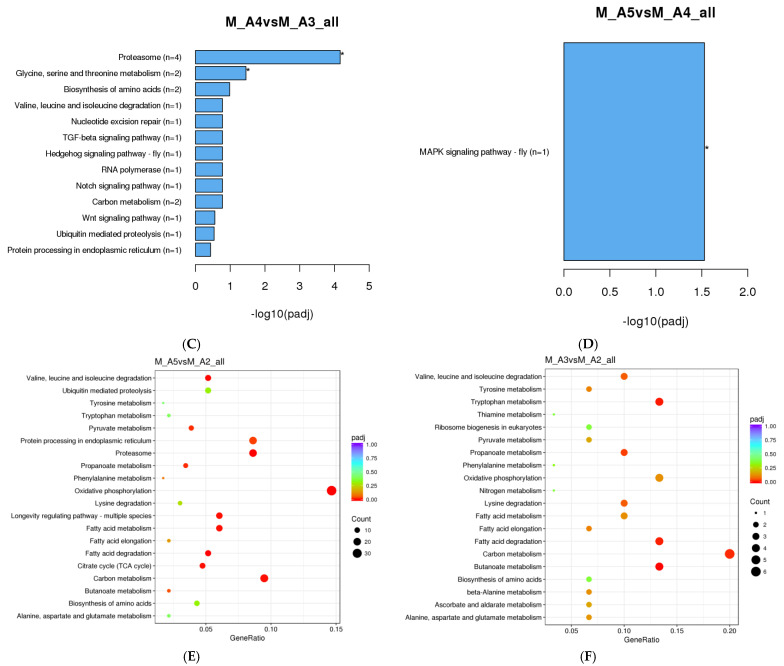

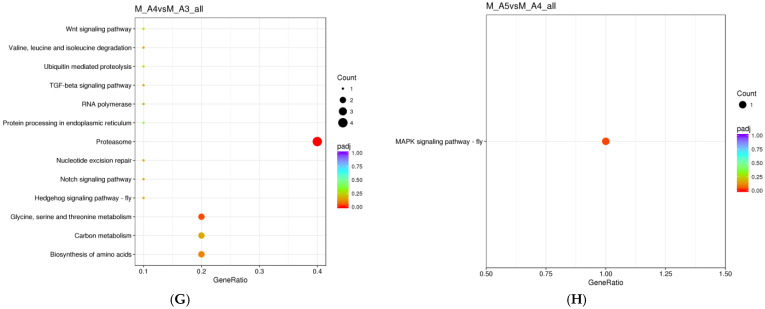
KEGG enrichment analysis of DEGs in male *Manduca sexta*. (**A**). Top enriched pathways comparing Age 2 and Age 5. (**B**). Top enriched pathways comparing Age 2 and Age 3. (**C**). Top enriched pathways comparing Age 3 and Age 4. (**D**). Top enriched pathway comparing Age 4 and Age 5. (**E**). Scatter plot of enriched pathways between Age 2 and Age 5. (**F**). Scatter plot of enriched pathways between Age 2 and Age 3. (**G**). Scatter plot of enriched pathways between Age 3 and Age 4. (**H**). Scatter plot of enriched pathway between Age 4 and Age 5. The *x*-axis represents the richness factor; the *y*-axis shows enriched pathway terms. * *p* < 0.05.

A KEGG analysis of male *M. sexta* revealed that the greatest amount of DEGs between middle age and advanced age moths were related to proteasomal complex elements (Figure 6A). Among male age classes, comparison of Age 2 and Age 3 moths displayed a majority of DEGs pertained to various metabolic pathways (Figure 6B). From Age 3 to Age 4, the greatest amount of DEGs were related to proteasomal complex elements (Figure 6C). KEGG analysis from Age 4 to Age 5 revealed only one enriched term pertaining to the MAPK signaling pathway (Figure 6D). A notable shift toward protein degradation pathways is evident when comparing Age 2 and Age 5 moths, as well as those transitioning from Age 3 to Age 4, aligning with the GO annotation results for these age classes. From Age 4 to Age 5 moths, there was one significantly enriched DEG pertaining to MAPK signaling. Intriguingly, this gene locus remains uncharacterized in *M. sexta* and its function (s) remains unclear.

**Figure 7:**
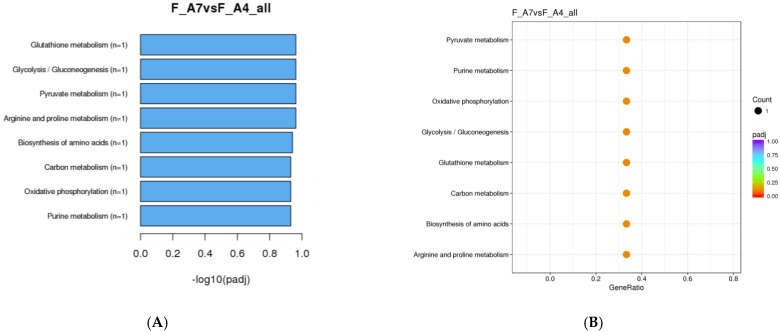
KEGG pathway enrichment analysis of DEGs in female *Manduca sexta.* (**A**). Top enriched pathway comparing Age 4 and Age 7. (**B**). Scatter plot of the top enriched pathways between Age 4 and Age 7. The *x*-axis represents the richness factor; the *y*-axis shows enriched pathway terms.

A KEGG analysis of female *M. sexta* comparing Age 4 and Age 7 revealed that the greatest number of DEGs were associated with metabolic pathways (Figure 7). However, there were no significantly enriched terms discovered. Intriguingly, there was no KEGG data available among progressive age classes. This is likely because there were no significant DEG transcript changes able to be identified in the data. KEGG analysis of female *M. sexta* provides further supporting evidence that few age-related changes are occurring in female moths as they progress from Age 4 to Age 7.

#### 3.1.4. Inventory of DEGs in Middle-Aged to Advanced-Aged Male M. sexta Flight Muscle

Significantly enriched DEGs (adjusted *p*-value < 0.05) were identified in male moths between Age 2 and Age 5. Log_2_ fold change (log_2_ FC) values were used to quantify expression differences. A log_2_ FC value of 1.0 for a given gene indicates a 2-fold increase in expression. Similarly, a log_2_ FC value of −2.0 represents a 4-fold decrease in expression (Table 5 and Table 6).

**Figure 8:**
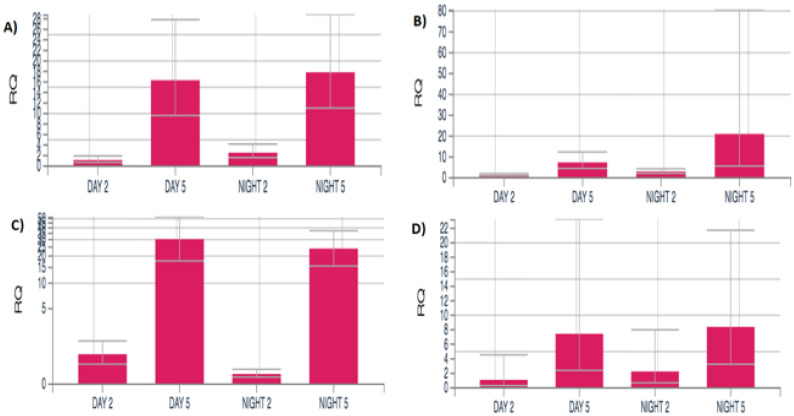
qRT-PCR expression levels of selected DEGs in male *Manduca sexta* comparing middle-aged and advanced-aged individuals across diel time. (**A**). *Akirin* (**B**). Gelsolin (**C**). Kelch (**D**). Titin.

RNA-Seq findings with *akirin* and titin, genes associated with muscle structural integrity, were corroborated by qRT-PCR (Figure 8).

#### 3.1.5. Progressive DEG Changes in Female Moths from Middle to Advanced Age

Few significant gene expression changes were detected in female moths across age classes (Table 7).

### 3.2. Metabolic Enzyme Activity

#### 3.2.1. HADH Activity

**Figure 9:**
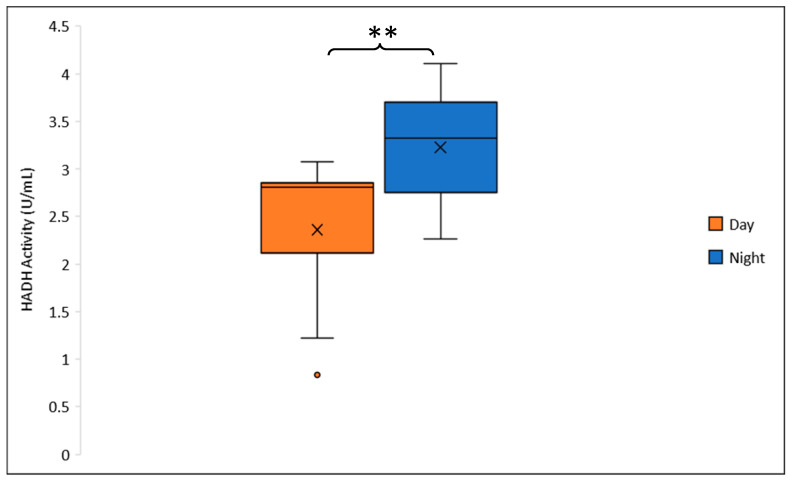
β-hydroxyacyl-CoA dehydrogenase (HADH) enzymatic activity in male *Manduca sexta* across diel time. Box plots represent the mean (solid line) and median (×), with boxes indicating the interquartile range (IQR). Whiskers denote the maximum and minimum values, and open circles represent outliers. Significance levels: ** *p* < 0.02.

An overall ANOVA revealed no significant difference in HADH activity between sexes (F_1,38_ = 0.128; *p* = 0.723). However, a three-way ANOVA indicated a significant diel effect in males: HADH activity was significantly higher in nighttime samples (*n* = 10) compared to daytime samples (*n* = 10) (Figure 9). On average, nighttime HADH activity in males increased by 26.8% relative to daytime levels (F_1,19_ = 6.967; *p* = 0.020). In contrast, no significant diel variation in HADH activity was observed in females (F_1,19_ = 0.374; *p* = 0.549). Additionally, HADH activity did not differ significantly across age groups in either males (F_1,19_ = 0.932; *p* = 0.349) or females (F_1,19_ = 0.374; *p* = 0.549).

#### 3.2.2. CS Activity

**Figure 10:**
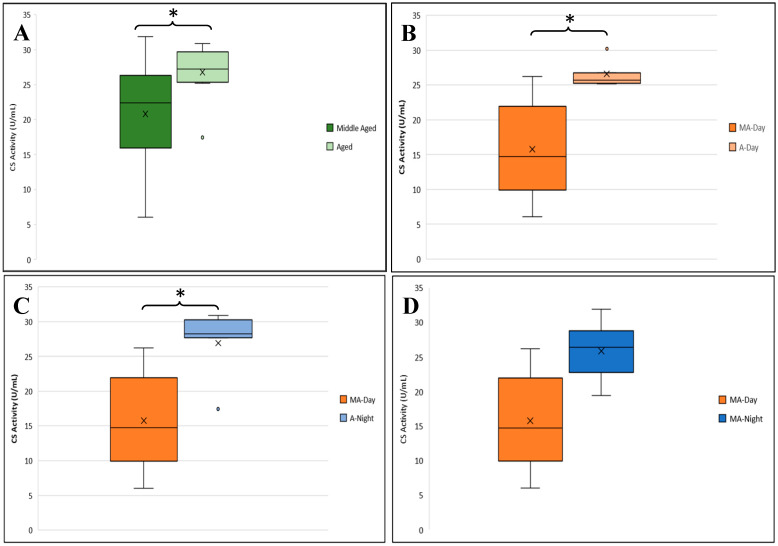
Citrate synthase (CS) activity in female *Manduca sexta*. (**A**). Mean CS activity across female age groups. (**B**). Comparison of CS activity between middle age (MA) and advanced age A females during the day. (**C**). Comparison of CS activity between middle-aged females during the day and advanced age females at night. (**D**). Comparison of CS activity between middle-aged females during the day and night. Significance levels: * *p* < 0.05.

An overall ANOVA showed a significant difference in CS activity between sexes (F_1,38_ = 4.454; *p* = 0.042). CS activity did not vary significantly by diel time in males (F_1,18_ = 0.518; *p* = 0.482) or females (F_1,18_ = 4.248; *p* = 0.055). No significant differences in CS activity were observed between male age groups (F_1,18_ = 0.011; *p* = 0.917). However, a three-way ANOVA revealed a significant difference in CS activity between middle-aged and aged female samples (Figure 10A). Specifically, CS activity was significantly lower in middle-aged females (*n* = 10, mean = 24.12) compared to aged females (*n* = 10, mean = 27.20).

A post hoc Tukey test identified specific group differences. CS activity differed significantly between middle-aged day and aged day female samples, as well as between middle-aged day and aged night samples (Figure 10B,C). The comparison between middle-aged day and night samples approached significance (*p* = 0.053; Figure 10D).

## 4. Discussion

Our study identified a critical age-associated transition in male *M. sexta*, with the most substantial transcriptional changes occurring between Age 2 and Age 3, marking this interval as a key inflection point in the decline of muscle and neuromuscular function. Time-series RNA-Seq analysis revealed that DEGs during this period were significantly enriched in pathways related to muscle structure, contractile function, and energy metabolism. Functional enrichment further highlighted pathways involved in protein catabolism, heat shock response, acetylcholine release, ion exchange, and mitochondrial oxidative stress mitigation. These transcriptional patterns suggest a shift toward increased proteolysis, reduced capacity to manage proteotoxic stress, and elevated acetylcholine signaling at neuromuscular junctions with advancing age. Together, these findings indicate a progressive deterioration of muscle homeostasis and neuromuscular integrity during the transition from middle to advanced age. Further investigation is warranted to elucidate the regulatory mechanisms driving these age-related transcriptional dynamics and their physiological consequences.

In contrast to males, female *M. sexta* exhibited minimal transcriptional changes across age classes, with few significant DEGs identified between Age 4 and Age 7. This likely reflects a younger biological age than expected, possibly due to extended lifespan under laboratory conditions. Females were unmated and housed separately, which might delay the onset of age-related transcriptional changes. This interpretation is consistent with previous studies [27,45,46] and aligns with known sex-specific feeding behaviors. Adult females feed during their nocturnal active period, while males typically do not feed post-eclosion, possibly contributing to their shorter lifespan.

The physiological and metabolic findings align with observed differences in nutrient utilization between sexes. Metabolically, females rely on nectar-derived fatty acids, and their increased fatty acid metabolism during activity suggests ongoing feeding [27]. In contrast, males show no significant change in long-chain fatty acid abundance over time, indicating a lack of nutrient intake post-eclosion. This feeding behavior likely enables females to maintain physiological homeostasis for longer periods, delaying the onset of age-related molecular decline. As a result, the female moths in this study might not have reached a sufficient biological age to exhibit progressive transcriptional aging, explaining the limited number of DEGs observed.

Together, these findings highlight the importance of accounting for sex-specific life history traits when interpreting transcriptomic aging data. Female *M. sexta*, which continue feeding throughout adulthood, might serve as a more representative model of normative aging, comparable to mammals and other continuously feeding species. In contrast, males, which cease feeding post-eclosion, might exhibit a more rapid or atypical aging trajectory. This distinction is crucial for accurately modeling and interpreting age-related molecular changes in insects and other organisms. Notably, our transcriptomic data from aging male *M. sexta* reveal molecular signatures strikingly similar to those observed in aged sarcopenic male mice, as reported by Kerr et al. [47], including declines in mitochondrial biogenesis, oxidative phosphorylation complexes, AMPK signaling, mitophagy, and stress response pathways, changes absent in aged female mice. Despite their non-feeding status, aging male moths appear to recapitulate key features of sarcopenia in mammalian systems, reinforcing *M. sexta* as an ideal invertebrate model for dissecting the molecular underpinnings of physiomuscular aging.

Building on this male-specific aging trajectory, transcriptomic and qRT-PCR analyses revealed a significant increase in titin gene expression from Age 2 to Age 5 in male *M. sexta*. Titin (also known as connectin) is a giant elastic filamentous protein in striated muscle sarcomeres, forming a third filament system alongside actin and myosin [41,42,48]. This protein functions as a molecular spring, generating passive tension during sarcomere extension [41,42,48]. The increased titin expression might reflect a shift in the cellular redox state [49,50,51], potentially triggering the mobilization of soluble titin to replace oxidatively damaged, non-functional titin within the sarcomere [52]. Beyond its mechanical role, titin is implicated in protein metabolism, myofibrillogenesis [42,53], proteasome localization, and the regulation of myofibril turnover [54]. These findings might indicate impaired protein quality control and disrupted muscle maintenance with age, contributing to sarcopenia and frailty. Our data, in conjunction with prior studies, suggest that elevated titin expression in aged muscle might signal dysregulation of titin homeostasis itself. This dysregulation could underlie a reduced capacity for muscle adaptation during aging, positioning titin as a key gene of interest in muscle aging. Further research is needed to clarify the role of titin in skeletal muscle aging and longevity pathways.

This age-associated titin increased expression highlights the broader remodeling of muscle tissue occurring in aging males. Complementing this, RNA-Seq and qRT-PCR data also revealed a significant increase in *Akirin* expression over the same age range, suggesting an additional layer of adaptive response through enhanced skeletal myogenesis. Akirin is a nuclear factor essential for innate immune responses [55] and is encoded by a highly conserved gene across vertebrates [56]. Skeletal muscle myogenesis is tightly regulated by both positive and negative growth factors [57]. Myostatin (GDF8), a negative regulator of muscle growth, is predominantly expressed in skeletal muscle and suppresses myogenesis. Loss-of-function mutations in myostatin result in increased muscle mass in mice and cattle, producing the “double muscling” phenotype [58,59,60]. Myostatin has also been shown to negatively regulate *Akirin* transcription [36,57,61]. Akirin promotes myogenic differentiation by acting on the p38 and PI3K pathways [57,62] and by inducing MyoD expression and IGF-II secretion [37,57], making it a critical promyogenic factor

As *Akirin* is a known pro-myogenic factor, its increased expression suggests activation of myogenic signaling pathways, which might represent a compensatory response to ongoing muscle degradation rather than a functional enhancement of myogenesis. In both rodents and humans, age-related muscle loss is characterized by reductions in muscle fiber number and cross-sectional area, largely due to impaired satellite cell function and diminished regenerative capacity [63,64]. Additionally, aging is associated with a shift toward a more oxidizing cellular redox environment across diverse taxa [49,50], which might exacerbate oxidative damage in skeletal muscle. Therefore, the elevated expression of *Akirin* might reflect a futile or dysregulated attempt at regeneration. Further investigation is required to elucidate the precise role of *Akirin* in age-associated muscle remodeling, its potential involvement in longevity pathways, and its relationship to the transcriptional patterns observed in this study.

In parallel with these promyogenic responses, RNA-Seq data also revealed significant age-related changes in the expression of genes involved in protein tagging, recognition, and degradation. DEGs encoding proteins and enzymes associated with protein catabolism showed marked upregulation, suggesting a systemic shift toward increased protein degradation with age. Specifically, expression of genes encoding components of the ubiquitin-proteasome system, including ubiquitin conjugation factor E4 B (*UBE4B*), 26S proteasome non-ATPase regulatory subunits (1, 2, 6, 7, 8, 11, 12, and 13), and 26S proteasome regulatory subunits 6A and 6B, was significantly elevated from middle to advanced age. These proteins are central to the ubiquitin-proteasome degradation pathway [65,66], indicating a probable global increase in proteolytic activity. Increased expression of ubiquitin carboxyl-terminal hydrolase isozyme L3 (*UCH-L3*) and Cullin-2, enzymes involved in deubiquitination and targeted protein degradation [67,68,69,70,71,72] further supports this trend.

Conversely, expression of several heat shock proteins (Hsps) declined with age, including Hsp68, αB-crystallin (HspB5), and lethal(2)-essential-for-life (l(2)efl). These proteins are critical for mitigating proteotoxic stress and refolding misfolded proteins. Hsp68, regulated by the JNK signaling pathway, plays a protective role against oxidative damage and is implicated in lifespan regulation [73,74,75]. Its downregulation suggests a diminished capacity for stress response and cellular repair. Alpha B-crystallin, a small heat shock protein expressed in skeletal and cardiac muscle, is involved in cytoskeletal stabilization, apoptosis regulation, and protein degradation [76,77,78]. Its reduced expression may contribute to muscle weakness and age-related myopathies, including muscular dystrophy and cardiomyopathy [79,80,81,82,83,84]. The (l(2)efl) gene, part of the Hsp20 family, also showed decreased expression across seven DEGs. In *Drosophila*, l(2)efl overexpression increases phosphorylation of eIF2α, suppressing protein synthesis [85]. Reduced l(2)efl expression in *M. sexta* may reflect a compensatory increase in protein synthesis in response to muscle degradation, though this remains speculative and warrants further investigation.

Additionally, most DEGs associated with metabolic pathways, particularly fatty acid β-oxidation, were decreased in aging males. This aligns with previous findings showing reduced fatty acid metabolism in aged *M. sexta* of both sexes [27]. A shift away from β-oxidation may indicate increased energy demands, altered mitochondrial function, or a metabolic transition toward glycolysis [86,87], potentially influenced by sex-specific, age-related physiomuscular changes [47].

This metabolic shift is likely exacerbated by post-eclosion fasting behavior in male moths. Insects rely on the fat body for nutrient storage and metabolism [88], and males depend on larval lipid reserves. By Age 5, these reserves are likely depleted, unlike in Age 2 individuals. This depletion might explain the observed reduced expression of β-oxidation genes, including *HADH* and insulin receptor genes, key components of the conserved insulin signaling pathway linked to aging [89,90,91,92,93,94].

Our data also revealed decreased expression of several ATPase subunits and antioxidant enzymes, including superoxide dismutase (*SOD*) and glutathione S-transferase 1-like, alongside increased expression of glutathione S-transferase 2-like. These patterns suggest reduced ATP production, elevated oxidative stress, and a compensatory upregulation of detoxification pathways. In mammals, the mitochondrial electron transport chain (ETC) is the primary source of ATP via oxidative phosphorylation, involving four transmembrane complexes and ATP synthase (F1F0-ATPase) [95,96]. Age-related declines in ETC efficiency may underlie the observed reductions in energy metabolism and increased oxidative damage in aging *M. sexta* muscle.

Although SODs directly neutralize ROS, evidence suggests that the cellular redox state shifts toward a more oxidizing environment with age across diverse taxa [49,50]. Interestingly, genetic manipulations that enhance cellular reducing capacity, rather than those that directly scavenge ROS have been more effective in extending lifespan [97,98]. For example, overexpression of glutathione S-transferases (*GST*s) in *Drosophila* has been shown to increase oxidative stress resistance and slow aging [99]. In *M. sexta*, *GST2*-like expression increased significantly from Age 2 to Age 5 in males, potentially reflecting a compensatory response to elevated ROS levels. These findings suggest that increased *GST* expression might serve as a mechanism to counteract oxidative damage during aging.

In parallel, RNA-Seq data revealed increased expression across numerous cell signaling-related DEGs. Notably, high-affinity choline transporters and vesicular acetylcholine (ACh) transporters were increased in expression, suggesting enhanced ACh release, a phenomenon also observed in aged mammals [100]. In mammals, neuromuscular function declines with age [101], and increased ACh release is hypothesized to compensate for reduced neuromuscular efficiency, helping to preserve muscle tone [102,103].

ACh also acts as an anti-synaptogenic agent at neuromuscular junctions (NMJs), promoting the disassembly of nicotinic ACh receptors (nAChRs) and regulating synaptogenesis [104,105,106,107]. In aged mice, increased cholinergic transmission has been linked to accelerated NMJ degeneration [108]. In *M. sexta*, increased expression of vesicular ACh transporters and high-affinity choline transporters from middle to advanced age suggests enhanced ACh storage and reuptake, mirroring mechanisms observed in mammals [109,110]. These findings support the hypothesis that aging in *M. sexta* involves increased cholinergic activity, potentially as a compensatory mechanism to maintain neuromuscular function.

## 5. Conclusions

Our comprehensive time-series transcriptomic analysis of aging male *M. sexta* muscle reveals a multifaceted molecular response to advancing age, characterized by significant changes in metabolism, stress response, and neuromuscular signaling. A pronounced increase in the expression of genes involved in protein catabolism, including components of the ubiquitin-proteasome system and deubiquitination enzymes, suggests a systemic shift toward enhanced protein degradation. Concurrently, elevated expression of structural genes such as titin, along with the pro-myogenic factor *Akirin*, may indicate ongoing structural remodeling and a compensatory activation of myogenic signaling pathways in response to muscle deterioration. However, given the well-established decline in regenerative capacity during muscle aging, particularly in vertebrate models, this expression pattern likely reflects a dysregulated or ineffective attempt at regeneration rather than a functional enhancement of myogenesis.

This apparent compensatory response is further complicated by a concurrent decline in cellular stress resilience. Specifically, the catabolic shift is accompanied by reduced expression of key heat shock proteins, including *Hsp68*, αB-crystallin, and *l(2)efl*, indicating a diminished capacity for proteostasis and stress adaptation. These changes might contribute to the onset of sarcopenia and frailty in aged moths. Additionally, a marked decrease in the expression of genes involved in fatty acid β-oxidation and ATP production, alongside increased markers of oxidative stress and antioxidant responses (e.g., *GST2*-like), reflects a metabolic transition likely driven by nutrient depletion and mitochondrial dysfunction.

In parallel, increased expression of vesicular acetylcholine and high-affinity choline transporters suggests enhanced cholinergic signaling at neuromuscular junctions, potentially as a compensatory mechanism to preserve muscle tone paralleling observations in aged mammalian models. Together, these findings highlight conserved molecular signatures of muscle aging and reinforce *M. sexta* as a valuable and well-suited complementary invertebrate model for investigating muscle aging, metabolic decline, and neuromuscular degeneration, owing to its physiological and metabolic similarities to mammalian muscle aging phenomena [28,111,112]. Critically, this system offers the potential for time-course molecular dissection spanning the entire physiomuscular aging process across the lifespan, from youth to advanced age, although the present study focused specifically on the transition from middle age to advanced age. Further research is warranted to elucidate the regulatory networks driving these changes and to clarify their implications for longevity and functional decline.

## Figures and Tables

**Table 1:** Age categories and sampling schedule for *Manduca sexta*.

Sex	Age Class	Sample Day (Post-Eclosion)	Diel Time and Age Sampling (D-Day and N-Night)
Male	Middle Age	Day 2 (D2)	D2, N2, D3, N3, D4, N4, D5, N5
	Advanced Age	Day 5 (D5)	
Female	Middle Age	Day 4 (D4)	D4, N4, D5, N5, D6, N6, D7, N7
	Advanced Age	Day 7 (D7)	

**Table 2:** Definitions and values of variables used in enzyme activity calculations.

Variable	Symbol	Value/Definition
Total Reaction Volume	*V*	0.2 mL
Dilution Factor	*dil*	11
Light Path	*d*	0.67 cm
Volume of Homogenate	*a*	0.002 mL
Molar Extinction Coefficient (CS)	*ϵ*	13.6 mM^−1^ cm^−1^ (TNB at 412 nm)
Molar Extinction Coefficient (HADH)	*ϵ*	6.22 mM^−1^ cm^−1^ (NADH at 340 nm)
Sample Absorbance (CS)	$A_{412}$	Absorbance (412 nm) minus background
Sample Absorbance (HADH)	$A_{340}$	Absorbance (340 nm) minus background

**Table 3:** Summary of RNA-Seq read counts and mapping statistics.

Sex	Raw Reads	Clean Reads	Mapping Efficiency (%)
Male	1,501,272,784	1,424,653,808	84.6
Female	1,520,863,862	1,437,381,790	87.2

**Table 4:** Forward and reverse primers designed for selected genes used in qRT-PCR.

Gene	Forward Primer	Reverse Primer
*Akirin*	5′–TTATGTTTCCCCACCTGTCTG	5′–GAACACAATTATCCAGCGAACC
Gelsolin	5′–TACATTCTGGACACGGGAAG	5′–TGAAACGTGTACCCAGTTAGG
Kelch	5′–TTC CTT GCT GTT CTC CCATAG	5′–AGTCCAAGTGTTTGTCCGTG
Titin	5′–TGAACCCTATTGAGTCTTGCTG	5′–GTGGCCTGACATGAAGTCTAG
Actin	5′–GCCAGAAAGACTCCTACGTTG	5′–TTCTCCATGTCATCCCAGTTG

**Table 5:** Inventory of significantly enriched differentially expressed genes between middle-aged and advanced-aged male *Manduca sexta*.

Pathway	Gene Name	log_2_ FC
Fatty Acid β-Oxidation	Trifunctional enzyme subunit alpha	−3.6287
	Acetyl-CoA acetyltransferase B	−2.4069
	Hydroxyacyl-coenzyme A dehydrogenase	−2.3855
	3-ketoacyl-CoA thiolase	−1.6528
	Probable enoyl-CoA hydratase	−1.3494
Tricarboxylic Acid (TCA) Cycle	Fumarate hydratase	−1.7915
	Succinate dehydrogenase [ubiquinone] flavoprotein subunit	−1.7434
	Succinate-CoA ligase [ADP-forming] subunit beta	−1.7418
	Pyruvate dehydrogenase E1 subunit alpha	−1.7222
	Malate dehydrogenase	−1.5403
	Citrate synthase 2	−1.4439
	Pyruvate dehydrogenase E1 subunit beta	−1.3942
Amino Acid Metabolism	2-oxoisovalerate dehydrogenase subunit beta	1.0183
	Methylcrotonoyl-CoA carboxylase beta chain	−1.8970
Oxidative Phosphorylation	NADH-ubiquinone oxidoreductase 75 kDa subunit	−8.7794
and Electron Transport Chain	NADH dehydrogenase [ubiquinone] iron-sulfur protein 6	−1.7617
	NADH dehydrogenase [ubiquinone] iron-sulfur protein 3	−1.5022
	NADH-ubiquinone oxidoreductase 49 kDa subunit	−1.2090
	Mitochondrial ATP synthase lipid binding protein	−1.7005
	ATP synthase subunit O	−1.6296
	ATP synthase subunit gamma	−1.5387
	ATP synthase subunit alpha	−1.3979
	ATP synthase subunit beta	−1.3196
	ATP synthase subunit d	−1.2778
	Cytochrome c1-2 heme protein	−1.6630
	Cytochrome b-c1 complex subunit 6	−1.3565
	Cytochrome b-c1 complex subunit 2	−1.3125
	Cytochrome c oxidase subunit 4 isoform 1	−1.3512
	Cytochrome c oxidase subunit 5B	−1.2896
Ubiquitin-Dependent Catabolism	Ubiquitin carboxyl-terminal hydrolase isoenzyme L3	2.0267
	Ubiquitin recognition factor in ER-associated degradation protein 1	1.7568
	Ubiquitin conjugation factor E4 B	1.6352
	Cullin-2	1.1491
	Ubiquilin-1	1.0763
	E3 ubiquitin-protein ligase siah2	−1.6271
Proteasome-Mediated Catabolism	Proteasome subunit alpha type 2	2.0653
	Proteasome subunit alpha type 7-1	2.0596
	26S proteasome non-ATPase regulatory subunit 8	1.8423
	Proteasome subunit alpha type 3	1.8211
	Proteasome subunit beta type 1	1.8012
	26S proteasome non-ATPase regulatory subunit 7	1.7898
	26S proteasome regulatory subunit 8-like	1.7408
	Proteasome subunit beta type 4	1.7372
	Proteasome subunit alpha type 1	1.7322
	26S proteasome non-ATPase regulatory subunit 13	1.6819
	26S proteasome regulatory subunit 10B	1.6822
	Proteasome subunit alpha type 4	1.6678
	UV excision repair protein RAD23 homolog A	1.6409
	26S proteasome regulatory subunit 7	1.6159
	26S proteasome non-ATPase regulatory subunit 1	1.599
	26S proteasome non-ATPase regulatory subunit 6	1.5746
	26S proteasome non-ATPase regulatory subunit 2	1.5629
	26S proteasome regulatory subunit 6A-B	1.536
	26S proteasome non-ATPase regulatory subunit 12	1.5142
	26S proteasome non-ATPase regulatory subunit 11	1.5134
	Peptide-N(4)-(N-acetyl-β-glucosaminyl)asparagine amidase	1.3655
Heat Shock Proteins	αB-crystallin	−3.3083
	Hsp68 (transcript 1)	−3.1492
	Hsp68 (transcript 2)	−1.971
	Lethal(2)-essential-for-life (l(2)efl) (7 transcripts)	−2.5424 to −1.7951
Oxidative Stress Response	Glutathione S-transferase 2-like	2.0604
	Muscle mitochondrial superoxide dismutase (SOD)	−1.8147
	Glutathione S-transferase 1-like	−1.0967
Muscle Structure	Titin (transcript 1)	2.6626
	Titin (transcript 2)	1.5671
	*Akirin*	1.3076
Other	Insulin receptor	1.4861
	Glucosamine-6-phosphate isomerase	1.4339

**Table 6:** Inventory of significantly enriched differentially expressed genes across progressive age classes in male *Manduca sexta*.

Age Comparison	Pathway	Gene Name	log_2_ FC
Age 2 vs. Age 3	Cellular Signaling	sodium- and chloride-dependent GABA transporter 1	2.3224
		sodium channel protein para	2.2053
		sodium/potassium/calcium exchanger 4	2.0749
		vesicular acetylcholine transporter	2.0226
		sodium/potassium/calcium exchanger Nckx30C	1.9863
		potassium voltage-gated channel unc-103	1.8663
		sodium/hydrogen exchanger 3	1.7188
		glycine receptor subunit alpha-4	1.6832
		high-affinity choline transporter 1	1.6273
		glutamate receptor 1	1.536
		GABA receptor subunit beta	1.5138
		sodium/calcium exchanger 1	1.3743
	Carbohydrate Metabolism	Facilitated trehalose transporter Tret1 (transcript 1)	2.1610
		Facilitated trehalose transporter Tret1 (transcript 2)	2.1029
Age 3 vs. Age 4	Calcium Signaling	Ryanodine Receptor	−1.3605

**Table 7:** Inventory of significantly enriched differentially expressed genes between middle-aged and advanced-aged female *Manduca sexta*.

Gene Name	log_2_ FC
Alsin	−1.7704
Sodium/hydrogen exchanger 3	−1.7424
Phosphate carrier protein	−1.327
Facilitated trehalose transporter	−1.1802
Ornithine decarboxylase	−1.1467

## Data Availability

Data will be made available on request.
